# Influence of metabolic syndrome and lifestyle factors on thyroid nodules in Chinese adult men: a cross-sectional study

**DOI:** 10.1530/ETJ-23-0168

**Published:** 2023-11-03

**Authors:** Ziyu Wan, Ying Li, Xiaoqian Dong, Yue Kang, Juan Luo, Jiangang Wang, Pingting Yang, Yaqin Wang, Yinglong Duan, Jianfei Xie, Andy S K Cheng

**Affiliations:** 1 Health Management Center, The Third Xiangya Hospital of Central South University, Changsha, Hunan, China; 2 Nursing Department, The Third Xiangya Hospital, Central South University, Changsha, Hunan, China; 3 Xiangya School of Nursing, Central South University, Changsha, Hunan, China; 4 Department of Rehabilitation Sciences, Hong Kong Polytechnic University, Hong Kong, China

**Keywords:** thyroid nodule, adult men, lifestyle, metabolic syndrome

## Abstract

**Introduction:**

Given the high prevalence of thyroid nodules and the potential for malignancy, it is imperative to understand the various factors that contribute to their development. This study aimed to explore the relationship between metabolic syndrome, lifestyle, and thyroid nodules in adult men in southern China.

**Methods:**

This study enrolled a total of 183,990 subjects at a medical examination center in a general hospital in southern China between January 1, 2015, and December 31, 2020. Multivariate logistic regression analysis was utilized to evaluate the relationship between metabolic syndrome, lifestyle factors, and thyroid nodules. Furthermore, structural equation modeling elucidated the intricate relationships among these variables.

**Results:**

The prevalence of thyroid nodules among Chinese adult males was 14.9%. Several factors were identified as risk factors for thyroid nodules, including advanced age, irregular meal time, smoking or quitting smoking, quitting drinking, heavy manual labor, hypertension, diabetes, dyslipidemia and centripetal obesity, and those belonging to ethnic minorities and drinking alcohol were found to be protective factors against thyroid nodules. Structural equation modeling highlighted metabolic syndrome's mediating role amidst lifestyle influences on thyroid nodules.

**Conclusion:**

The prevalence of thyroid nodules in Chinese adult males is relatively moderate to low. The factors identified in this study can help clinicians identify high-risk patients and develop targeted screening strategies for the timely detection of thyroid nodules. However, further mechanistic research and longitudinal studies are necessary to explore the underlying causes and establish causal relationships.

## Introduction

Thyroid nodules (TNs) refer to discrete lesions within the thyroid gland and are one of the most common clinical thyroid disorders. Most TNs are occult, with physical examination revealing them in only 5% of patients (1). However, the incidence of TNs is reported to be between 10.2% and 38.4% (2, 3, 4, 5), and up to 7.9% of these nodules are malignant (6). Recent advancements in ultrasonographic techniques have enabled the early detection of TNs (7), thereby providing an effective tool to investigate factors that influence their occurrence. Given the high prevalence of TNs and the potential for malignancy, it is imperative to understand the various factors that contribute to their development. This knowledge can help clinicians identify at-risk groups, leading to early detection and treatment of TNs.

Several studies have reported a positive correlation between metabolic syndrome (MetS) and the risk of TNs (8, 9, 10). MetS is a pathological condition that includes abdominal obesity, insulin resistance, hypertension, and hyperlipidemia (11). MetS is more severe in developing countries due to unhealthy dietary patterns and reduced exercise (11). The association of MetS with the incidence of TNs provides evidence for identifying patients at risk. After stratification by gender, individual symptoms of MetS can be independent risk factors for TNs in women (12, 13, 14), whereas the association remains controversial in men (13, 15). Further studies are required to clarify the association between MetS and TNs in men.

In addition to MetS, lifestyle factors have also been identified as influential factors for TNs. Studies have shown that work physical intensity, smoking, and alcohol consumption (16, 17), as well as dietary habits (18, 19), have different effects on the development of TNs. However, these studies have small sample sizes, inconsistent reported incidence rates, unknown specific mechanisms, and even conflicting results, making it difficult to draw definitive conclusions. At the same time, lifestyle has been also identified as a determinant of MetS (20, 21). Nonetheless, predominant studies investigating the etiological factors of TNs tend to position both MetS and lifestyle as parallel factors, thereby neglecting the intrinsic linkage that exists directly between lifestyle and the onset of MetS.

The purpose of this study is to conduct a cross-sectional investigation of 183,990 adult men in southern China in order to examine the prevalence of TNs and to determine their association with MetS and lifestyle, especially considering the mediating role of MetS in the influence of lifestyle on the occurrence of TNs.

## Materials and methods

### Participates

This study used convenience sampling and included male participants who underwent physical examinations at a health management center of a general hospital in southern China from January 1, 2015, to December 31, 2020. A total of 241,389 individuals completed the medical examinations. Participants under 18 years of age, those who refused or failed to complete dietary diversity testing, and those who underwent MetS index testing or thyroid ultrasound, and had a history of pre-existing thyroid disease, such as thyroid dysfunction or thyroid cancer, were excluded from the study. After exclusions, a total of 183,990 participants were included in the final analysis. The informed consent of all participants has been obtained according to the requirements of the hospital ethics committee.

### Demographic characteristics and basic Information

Demographic information and anthropometric parameters were measured and recorded by trained nurses. These parameters included age, nation, working intensity, regular meal time, smoking and drinking alcohol, drinking coffee, and exercise.

We measured patients’ weight (in kilograms), height (in meters), systolic blood pressure (SBP), and diastolic blood pressure (DBP). And a tape measure was used to gauge waist circumference (WC) past the midpoint between the upper edge of the iliac crest and the lower edge of the rib cage.

### Laboratory examination

After the subject had fasted for 12 h, a venous blood specimen is drawn for laboratory examination to test fasting blood glucose (FBG), glycated hemoglobin (HbA1C), serum triglyceride (TG), and high-density lipoprotein cholesterol (HDL-C).

### Ultrasound examination

Subjects were placed in a supine position with the neck exposed, and thyroid ultrasonography was performed by a specialist ultrasonographer. TNs were characterized as discrete lesions within the thyroid gland, distinguishable from the surrounding thyroid tissue through radiological features. A diameter greater than 3 mm is the criterion for determining the presence of a TN.

### Definition of variables

Exercise: Participants’ ‘exercise’ behaviors were gauged based on their reported activities from the preceding week. Those who engaged in physical activities for at least 30 min on more than three occasions during that week were classified as ‘actively participating in exercise’. If not, they were categorized as ‘not or rarely participating in exercise’ (22).

Regular meal time: ‘Regular meal time’ was relied on the participants’ subjective evaluations. They were prompted to designate their regular eating patterns as either ‘regular’ or ‘irregular’, according to their personal routines and perceptions.

Working intensity: On the basis of the documents issued by SBQTS (the state bureau of quality and technical supervision) of China (23), we added the category of no working and divided the work intensity into four categories: ‘no working’, ‘office workers’, ‘light manual laborers’, and ‘medium and heavy manual laborers’ according to physical strength.

Hypertension: According to the Revision Committee of Chinese Guidelines for Prevention and Treatment of Hypertension, hypertension was classified as high SBP/DBP ≥ 140/90 mm Hg (1 mmHg = 0.133 kPa) or taking antihypertensive drugs (24).

Diabetes: According to the American Diabetes Association, participants were diagnosed with diabetes when their FBG ≥ 7.0 mmol/L or HbA1C ≥ 6.5% or when they took hypoglycemic drugs. The standard of pre-diabetes is 5.6 ≤ FBG < 7.0 or 5.7% ≤ HbA1C < 6.5% (25).

Dyslipidemia: International Diabetes Federation defines dyslipidemia as TG ≥ 1.7 mmol/L or HDL-C < 1.03 mmol/L (26).

Obesity: Obesity is defined in four classes according to WC and BMI: no obesity (BMI<28, WC<90 cm), BMI obesity (BMI ≥ 28, WC<90 cm), abdominal obesity (BMI<28, WC ≥ 90 cm), and combined obesity (BMI ≥ 28, WC ≥ 90 cm) (27).

Dietary diversity score (DDS): Subjects were asked to review the types of food they consumed during the week. And all foods were divided into nine categories: grains (including roots and stems), vegetables, fruits, meat (including pork, beef, poultry, and animal parts), legumes (including nuts), eggs, fish (including all types of fish), dairy products, and oils (animal and vegetable oils). Each food consumed is counted as one point, and the total score is 9. The consumption of different kinds of food from the same category is not counted repeatedly. It is classified as DDS-1 (1–3 points), DDS-2 (4–6 points), and DDS-3 (7–9 points) according to the purpose of this study (28, 29). However, due to the limited representation of DDS-1 in our dataset (1.4%), we merged DDS-1 and DDS-2 into a combined category labeled ‘Poor and average diversity’. DDS-3 was labeled as ‘Rich diversity’.

### Statistical analysis

Data analysis was performed using Statistical Package for the Social Sciences 25. Continuous variables were described by mean ± s.d. and categorical variables were presented by counts and percentages. Differences in TN prevalence between groups were assessed using the chi-square test, while differences in the means of continuous variables between the TN group and the normal group were assessed using *t*-tests. Variables with a *P*-value of <0.05 were eventually included in multivariate binary logistic regression analysis. The assessment for multicollinearity was conducted using the Variance Inflation Factor (VIF). A VIF value of 5 or greater is indicative of the presence of multicollinearity. Dummy variables were created for ‘working intensity’, ‘smoking habits’, ‘drinking habits’, ‘diabetes’ and ‘obesity’ when performing logistic regression. All tests were two tailed. Significant *P* was taken as 0.05.

**Figure 1:**
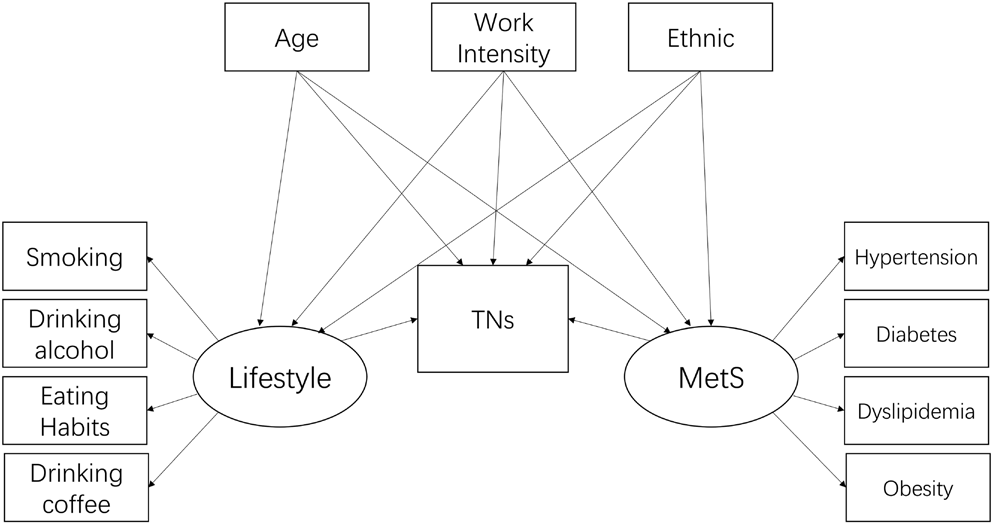
Base structural equation model.

Following our logistic regression analysis, we incorporated the significant findings into a structural equation model (SEM) to explore mediation effects. Drawing insights from a comprehensive literature review, we proposed an initial research model as depicted in Fig. 1. We utilized R (version 4.3.1) for the SEM analysis and adopted the Weighted Least Squares Mean and Variance Adjusted (WLSMV) method. This method is particularly apt for SEM when dealing with categorical and ordinal observed variables. Given the limited interpretative power of the chi-square value in large samples (30), we relied on three key fit indices to assess the model’s fit: Tucker–Lewis index (TLI ≥ 0.90), Comparative fit index (CFI ≥ 0.90), and root mean square error of approximation (RMSEA ≤ 0.05) (31).

## Results

### Demographic characteristics and single-factor analysis

As shown in Table 1, the prevalence of TNs was 14.9% among the 183,990 Chinese adult males included in the study. The mean age of the subject population was 44.9 ± 13.3, with a predominance of Han Chinese (96.8%). Most participants had a regular diet (77.6%). More than half of the participants reported being non-smokers (51.9%) and non-drinkers (50.4%). Two-thirds of the adult males participated in sports (67.3%). In terms of work physical strength, 66.2% of adult males were engaged in mental work. As for MetS, the prevalence of hypertension was 23.6%, prediabetes was 27.9%, diabetes was 8.5%, and dyslipidemia was 51.6%. In addition, 19.7% of participants had abdominal obesity, while 14.1% had both abdominal and BMI obesity.

**Table 1:** Baseline data and results of single-factor analysis.

	Total	non-TNs	TNs	*χ*^2^/*t*	*P*
Total	183,990	156,589 (85.1)	27,401 (14.9)		
Age	44.88 ± 13.31	43.80 ± 13.15	51.08 ± 12.54	88.00	<0.001
SBP (mmHg)		125.40 ± 14.53	127.98 ± 15.83	28.77	<0.001
DBP (mmHg)		77.80 ± 10.67	79.48 ± 10.97	26.71	<0.001
BMI		24.90 ± 3.14	25.29 ± 3.00	19.49	<0.001
WC		85.94 ± 8.61	87.22 ± 8.32	26.60	<0.001
FBG		5.57 ± 1.35	5.84 ± 1.64	26.23	<0.001
HbA1C		5.68 ± 0.86	5.80 ± 0.99	11.26	<0.001
TG		2.11 ± 1.95	2.14 ± 1.91	2.71	0.007
HDL-C		1.25 ± 0.28	1.24 ± 0.27	6.34	<0.001
Age (graded)				7599.05	<0.001
19–29	21,797 (11.9)	20,756 (13.3)	1041 (3.8)		
30–39	50,593 (27.5)	46,408 (29.6)	4185 (15.3)		
40–49	46,924 (25.5)	40,080 (25.6)	6844 (25.0)		
50–59	39,054 (21.2)	30,289 (19.3)	8765 (32.0)		
60+	25,622 (13.9)	19,056 (12.2)	6566 (24.0)		
Ethnicity				73.97	<0.001
Han	178,046 (96.8)	151,298 (96.6)	26,748 (97.6)		
Minorities	5944 (3.2)	5291 (3.4)	653 (2.4)		
Regular meal time				856.54	<0.001
Eating on time	142,747 (77.6)	123,352 (78.8)	19,395 (70.8)		
Not eating on time	41,243 (22.4)	33,237 (21.2)	8006 (29.2)		
DDS				0.003	0.956
Poor and average	103,580 (56.3)	88,150 (56.3)	15,430 (56.3)		
Rich	80,410 (43.7)	68,439 (43.7)	11,971 (43.7)		
Drinking coffee				91.75	<0.001
No	131,656 (71.6)	111,389 (71.1)	20,267 (74.0)		
Yes	52,334 (28.4)	45,200 (28.9)	7134 (26.0)		
Smoking habits				134.29	<0.001
No Smoking	95,471 (51.9)	82,100 (52.4)	13,371 (48.8)		
Smoking	77,957 (42.4)	65,715 (42.0)	12,242 (44.7)		
Quit smoking	10,562 (5.7)	8774 (5.6)	1788 (6.5)		
Drinking habits				98.31	<0.001
No Drinking	92,814 (50.4)	79,048 (50.5)	13,766 (14.8)		
Drinking	87,255 (47.4)	74,422 (47.5)	12,833 (46.8)		
Quit Drinking	3921 (2.1)	3119 (2.0)	802 (2.9)		
Exercise				149.64	<0.001
No exercise	60,111 (32.7)	52,035 (33.2)	8076 (29.4)		
Exercise	123,879 (67.3)	104,554 (66.8)	19,325 (70.6)		
Working intensity				805.05	<0.001
Non-worker	11,691 (6.5)	9154 (5.8)	2807 (10.2)		
Office worker	121,871 (66.2)	104,978 (67.0)	16,893 (61.7)		
Light manual laborer	36,179 (19.7)	30,647 (19.6)	5532 (20.2)		
Medium and heavy manual laborer	13,979 (7.6)	11,810 (7.5)	2169 (7.9)		
Hypertension				1512.52	<0.001
Normal	140,589 (76.4)	122,173 (78.0)	18,416 (67.0)		
Hypertension	43,401 (23.6)	34,416 (22.0)	8985 (32.8)		
Diabetes				1492.51	<0.001
Normal	116,956 (63.6)	102,099 (65.2)	14,857 (54.2)		
Prediabetes	51,265 (27.9)	42,324 (27.0)	8941 (32.6)		
Diabetes	15774 (8.5)	12,169 (7.8)	3605 (13.1)		
Dyslipidemia				40.42	<0.001
Normal	89,005 (48.4)	76,235 (48.7)	12,770 (46.6)		
Dyslipidemia	94,985 (51.6)	80,354 (51.3)	14,631 (53.4)		
Obesity				549.88	<0.001
Normal	119,989 (65.2)	103,745 (66.3)	16,244 (59.3)		
BMI obesity	1923 (1.0)	1674 (1.1)	249 (0.9)		
Centripetal obesity	36,216 (19.7)	29,662 (18.9)	6554 (23.9)		
Combined obesity	25,862 (14.1)	21,508 (13.7)	4354 (15.9)		

DBP, diastolic blood pressure; DDS, dietary diversity score; FBG, fasting blood glucose; HbA1C, glycated hemoglobin; HDL-C, high-density lipoprotein cholesterol; SBP, systolic blood pressure; TG, serum triglyceride; WC, waist circumference.

As shown in Table 1, TNs group had higher age (*P* < 0.001), SBP (*P* < 0.001), DBP (*P* < 0.001), BMI (*P* < 0.001), WC (*P* < 0.001), FBG (*P* < 0.001), HbA1C (*P* < 0.001), TG (*P* = 0.012), and lower HDL-C (*P* < 0.001) than non-TNs group.

### Logistic regression analysis of TNs’ risk factors

**Figure 2:**
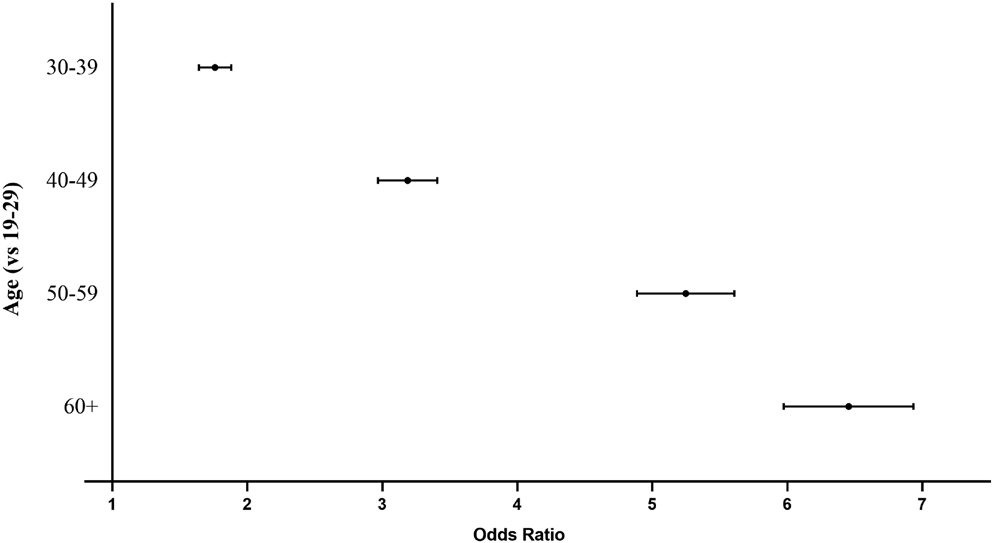
Forest plot of thyroid nodules’ influence factors (age).

**Figure 3:**
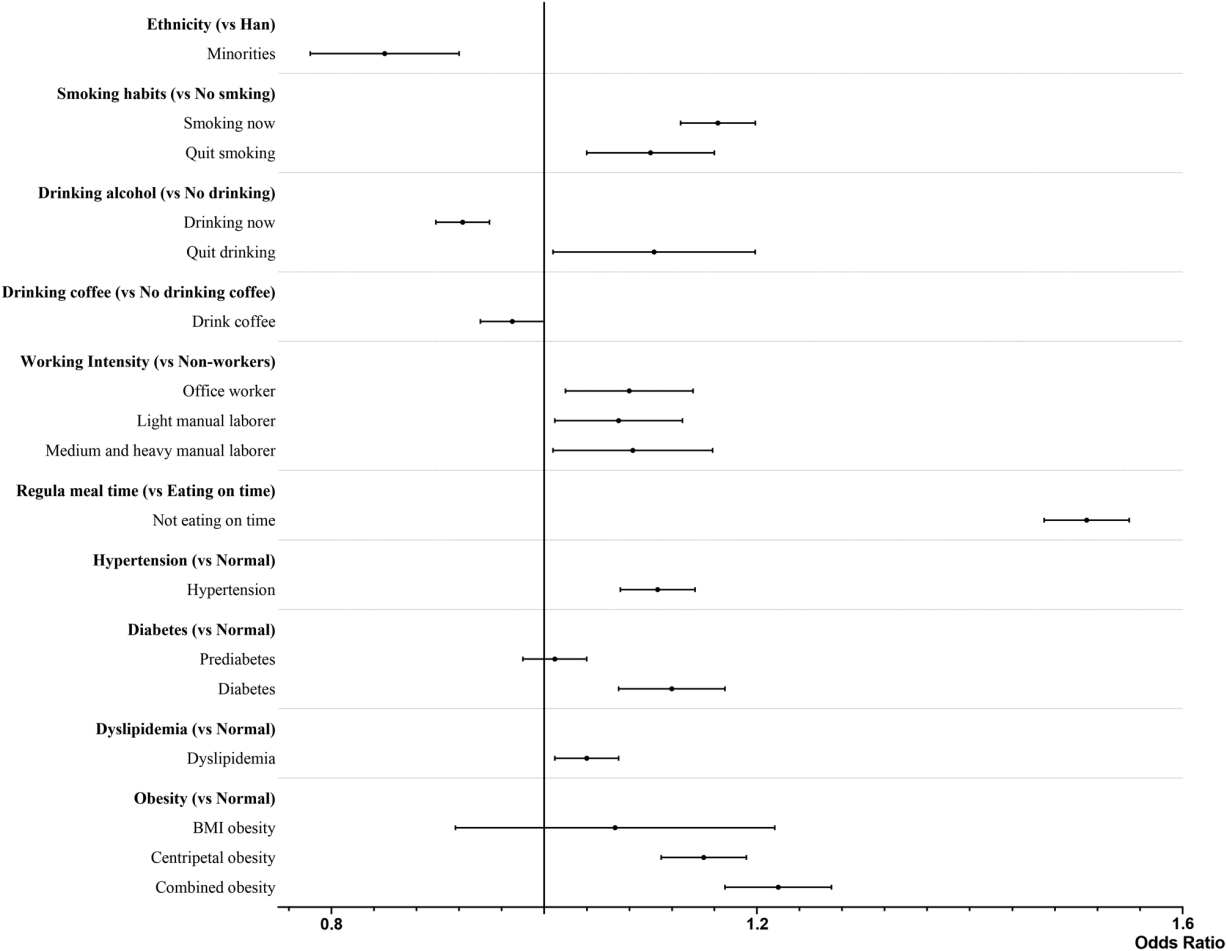
Forest plot of thyroid nodules’ influence factors (others).

The model was built after verifying the multicollinearity. Upon comparison with the baseline model, which had a Nagelkerke *R*^2^ of 0.033, we opted for an alternative model that incorporates key covariates: age, ethnicity, and work intensity. The refined model demonstrated enhanced performance, evidenced by a –2 log-likelihood value of 145,782.12 and a Nagelkerke *R*^2^ of 0.085. As shown in Figs. 2 and 3, among adult men, advanced age (OR = 6.44, 95% CI: 5.98–6.93), irregular meal time (OR = 1.51, 95% CI: 1.47–1.55), smoking (OR = 1.16, 95% CI: 1.13–1.20) or quit smoking (OR = 1.10, 95% CI: 1.04–1.16), quit drinking (OR = 1.10, 95% CI: 1.01–1.20), hypertension (OR = 1.11, 95% CI: 1.07–1.14), diabetes (OR = 1.12, 95% CI: 1.07–1.17), dyslipidemia (OR = 1.04, 95% CI: 1.01–1.07), centripetal obesity (OR = 1.15, 95% CI: 1.11–1.19), and combined obesity (OR = 1.22, 95% CI: 1.17–1.27) were positively correlated with the presence TNs. Ethnic minorities (OR = 0.85, 95% CI: 0.78–0.92), drinking coffee (OR = 0.97, 95% CI: 0.94–0.99), and drinking alcohol (OR = 0.92, 95% CI: 0.90–0.95) were inversely associated with the presence of TNs.

### SEM model of factors associated with TNs

**Figure 4:**
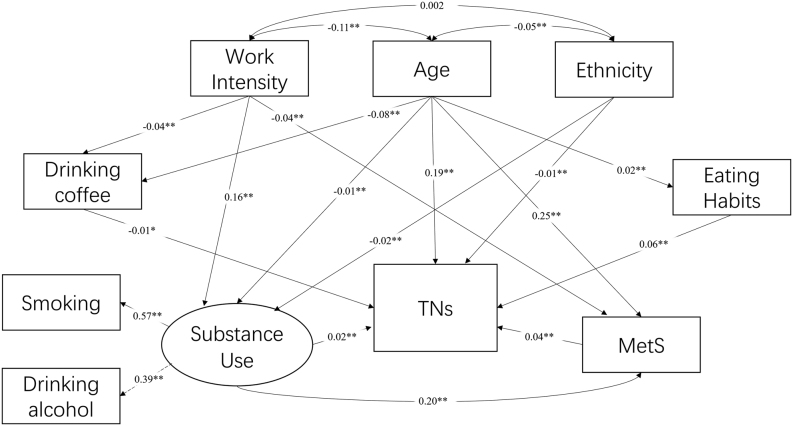
Final structural equation model.

Figure 4 displays the refined SEM model. Based on the outcomes of the logistic regression, we optimized the foundational model by eliminating non-significant paths and omitting observed variables with weak factor loadings. Notably, due to their non-significant loadings, both ‘drinking coffee’ and ‘regular meal time’ were dissociated from the latent variable previously labeled ‘lifestyle’ and were subsequently treated as individual mediators. As a result, the ‘lifestyle’ latent variable was aptly renamed to ‘substance use’. To further enhance the model’s fit, the latent variable ‘MetS’ was substituted with the count of MetS symptoms. For a detailed overview of the final eight observed variables retained in the model, readers are referred to Supplementary Material 1 (see section on supplementary materials given at the end of this article). All path coefficients and loadings in the figure are standardized. The data exhibits a robust alignment with our measurement model, as evidenced by the fit indices: CFI = 0.957, TLI = 0.910, and RMSEA = 0.027.

In the final SEM model, it was evident that within the realm of lifestyles, drinking coffee emerged as a protective element against TNs (–0.01, *P* < 0.001). On the contrary, behaviors such as not having regular meal times (0.06, *P* < 0.001), substance abuse (0.02, *P* < 0.001), and presenting with an increased number of MetS symptoms (0.04, *P* < 0.001) were associated with heightened risks of TNs. And the chi-square value for TNs was determined to be 0.046. Table 2 presents the direct and indirect effects of the research variables on TNs. Notably, within the trajectory where lifestyle exerts its impact on TNs, the intermediary role of MetS was pronouncedly significant.

**Table 2:** Direct, indirect, and total effects of variables on TNs.

Variables	Direct	Indirect	Total
Age	0.190	0.012	0.202
Work intensity	No path	0.003	0.003
Ethnic	−0.009	−0.001	−0.010
Drinking coffee	−0.008	No path	−0.008
Eating habits	0.064	No path	0.064
Substance use	0.020	0.007	0.027
MetS	0.036	No path	0.036

MetS, metabolic syndrome.

## Discussion

The prevalence of TNs is increasing every year, which underscores the need for greater attention. In this study, the prevalence of TNs in Chinese adult males was 14.9% and falls within the moderate-to-low range reported in other similar studies conducted in China (2, 3, 4). These disparities may be attributed to differences in the lifestyle and dietary habits of the local population (4). Using SEM to study the complex relationship among lifestyle, MetS and TNs is the innovation of this study. We found that the risk of TNs in Chinese adult men was positively associated with advanced age, MetS (obesity, hypertension, diabetes, and dyslipidemia), and lifestyle (smoking, non-drinking alcohol, and irregular meal timing).

Previous studies have identified advanced age as a recognized risk factor for MetS and TNs (4, 8, 32). In this study, age directly or indirectly affects the incidence of TNs through multiple pathways. The univariate analysis and logistic regression report the same results: TNs group had a significantly higher mean age than non-TNs group (51.1 vs 43.8 years). Participants over 60 years old had a 6.53 times higher risk of developing TNs compared to those in the 19–29-year-old age group. According to the prevailing belief, the impact of environmental factors on the human body intensifies over time. This impact is concomitant with a decline in the body’s immune competence, which leads to the impairment of thyroid cells due to oxidative stress by free radicals. As a result, these pathological events culminate in thyroid fibrous connective tissue hyperplasia and inflammatory infiltration, ultimately leading to the emergence of TNs (33, 34).

The present study discovered significant disparities in TNs prevalence between Han and ethnic minority populations in southern China. In the logistic regression, ethnic minorities were found to have a 15% lower likelihood of TNs than Han adult males. The direct influence of ethnicity on TNs might stem from genetic variations, and the mediating effect arises through its impact on substance use. It is worth mentioning that different ethnic groups often have distinct lifestyles and dietary patterns. Moreover, the ethnic minorities in Hunan Province are concentrated in the Xiangxi Tujia and Miao Autonomous Prefecture, an area characterized by low urbanization and mountainous forested regions, which could have implications for thyroid health. However, it is important to note that the sample size of ethnic minorities included in this study was relatively small (3.2%) and there is a lack of relevant literature to confirm these inferences. Further in-depth studies are required to investigate the underlying reasons for these differences, including increasing the sample size of ethnic minorities and carefully classifying different minority groups.

In relation to dietary habits, our study unveils a unique insight: not eating on a regular time emerges as a potential risk factor for the development of TNs – a finding that has yet to be reported in existing literature. Specifically, participants who did not maintain consistent meal schedules were found to have a 1.51 times greater risk of developing TNs compared to their counterparts who ate regularly. On a different note, coffee consumption appeared to act as a protective agent against TNs. Current research suggests that coffee might help sustain thyroid-stimulating hormone (TSH) levels within a healthy range (35) or reduce inflammation through specific coffee extract (36), which could contribute positively to the thyroid gland’s structure and functionality. However, it is pivotal to recognize the disparities inherent in Chinese and Western coffee-drinking cultures. Coffee was introduced to China relatively late, and Chinese individuals tend to consume coffee primarily for specific purposes, such as alleviating fatigue and staying alert (37). These habits may be associated with unhealthy practices like staying up late and insufficient sleep. However, these lifestyle factors were not included in the current study, leaving a gap in our understanding.

The logistic regression analysis demonstrated that participation in exercise was not found to be an independent risk factor for TNs; however, working intensity was shown to have a significant association with TNs. The likelihood of TNs in office workers, light-to-heavy laborers is 1.07–1.08 times that of non-workers. According to Dong *et al.*’s study, there is a positive correlation between physical intensity at work and the prevalence of TNs (12). Instead, the effect was mediated through three pathways: coffee consumption, substance use, and MetS. Those engaged in more physically demanding jobs tend to smoke or consume alcohol more frequently and are less likely to drink coffee, which has been suggested to have protective effects against TNs. Interestingly, in our study, these physically active workers also showed a lower propensity for MetS. Additionally, some researchers posit that heavy physical labor can lead to heightened oxidative stress and inflammation (38). Such adverse responses could potentially have detrimental effects on both TNs and MetS. Therefore, there is a pressing need for more mechanistic studies to delve into the relationship between physical labor and the onset of TNs.

In this study, substance use was correlated with a higher risk of TNs. Smoking and alcohol consumption were the observed variables that make up the latent variable ‘substance use’. The effects of smoking and drinking on TNs remain a matter of debate. Smoking was found to be an independent risk factor for TNs in logistic regression. Both current and former smokers showed a higher incidence of TNs compared to non-smokers. These findings are in line with the results of Jiang *et al.’*s study, which demonstrated that harmful substances present in tobacco smoke can adversely affect the growth and differentiation of thyroid cells (39). Moreover, smoking can also lead to disruptions in TSH levels (40), which may contribute to the TNs’ development. However, this conflicts with the prevailing view. A cohort study of 90,000 Korean adults by Cho *et al.* found that smoking is a protective factor for TNs (41). They suggested that smoking may reduce TSH levels and alkaloids contained in tobacco may also reduce the incidence of autoimmune thyroiditis (17). We also found that the likelihood of TNs was lower in drinkers and higher in abstainers compared to non-drinking adult males. This finding is in line with a systematic review by Balhara *et al.* (42). Current evidence suggests that alcohol has a direct toxic effect on thyroid cells, leading to thyroid suppression and a reduction in the size of the thyroid gland (42). Furthermore, chronic alcohol exposure has been shown to impair the normal response of the hypothalamic–pituitary–thyroid axis to central stimuli, as previously reported (16, 43). Specifically, alcohol impact the response of the hypothalamic–pituitary–thyroid axis to central stimuli, such as thyrotropin-releasing hormone (TRH). This can ultimately affect TSH secretion and thyroid function. Interestingly, upon alcohol withdrawal, this process can be reversed and TSH levels may be re-elevated. However, there are currently no consensus regarding how smoking and drinking alcohol affects TNs and further mechanistic research is necessary to investigate.

Structural equation modeling revealed that MetS played a pivotal mediating role in the influence of various lifestyle factors on TNs. Specifically, an increase in the number of MetS symptoms was linked to a heightened risk of TNs. These findings are consistent with a study by Shin *et al.* (2), which demonstrated that individuals with multiple MetS manifestations are at a higher risk of developing TNs compared to those with no or only one MetS manifestation. Logistic regression also reports similar results, identified several components of MetS, including hypertension, diabetes, dyslipidemia, and obesity as independent risk factors for TNs.

In addition, this study revealed that central obesity was strongly linked to the presence of TNs, whereas the association between BMI obesity and TNs was not significant. The lack of significance with BMI obesity may be due to its composite nature, which takes into account not only fat mass but also muscle, bone mass, and height. In contrast, central obesity demonstrated by WC focuses on abdominal fat deposition. Central obesity is more closely associated with poor metabolism (44). Obesity is positively correlated with insulin resistance, which affects the proliferation and differentiation of thyroid cells through insulin secretion and is significantly associated with the formation of TNs (14, 15). In addition, obesity is associated with chronic low-grade inflammation, which produces inflammatory markers that can induce changes in thyroid vascular permeability and even lead to autoimmune responses, ultimately resulting in morphological or functional changes in the thyroid gland (45). Our findings hint at a potential relationship between weight management, particularly concerning abdominal obesity and TNs.

However, several limitations of this study should be considered. First, the primary focus of this study was on the presence of TNs, without an in-depth exploration of their specific attributes. As a result, it provides a somewhat limited perspective. Second, the study was conducted at a single hospital in southern China, which may limit its generalizability to other regions or populations. Third, the impact of iodine intake was not taken into account in the collection of dietary information. Fourth, laboratory tests for thyroid hormones such as TSH were not conducted, which could have provided additional insights into the relationship between MetS and thyroid function. These limitations highlight the need for further research using more robust study designs, broader populations, and more comprehensive data collection methods to fully understand the relationship between MetS, lifestyle, and TNs.

## Conclusion

In this study, the prevalence of TNs in Chinese adult males was 14.9%. Advanced age, smoking or quit smoking, quit drinking, heavy working, irregular meal time, hypertension, diabetes, dyslipidemia, centripetal obesity, and combined obesity were risk factors for TNs. Drinking coffee, ethnic minorities, and drinking alcohol were protective factors for TNs. The study emphasizes the importance of promoting healthy eating habits, drinking or smoking, weight management, and workload factors to reduce the risk of TNs.

## Supplementary Materials

Supplementary Material 1. Overview of the final observed variables

## Declaration of interest

The authors declare that there is no conflict of interest that could be perceived as prejudicing the impartiality of the research reported.

## Funding

This study is sponsored by the Special Funding for the Construction of Innovative Provinces in Hunan (NO.2020SK53618). The funding support provided covered the research personnel’s labor fees, data storage and management expenses, transportation costs, and expenses incurred during the publication process of the paper.

## Statement of ethics

This study protocol was reviewed and approved by Ethics Committee of The Third Xiangya Hospital of Central South University, approval number 2020-S587. And the written informed consent of all participants has been obtained according to the requirements of the hospital ethics committee.

## Data availability statement

Data queries can be directly sent to corresponding author.

## Author contribution statement

ZW: conceptualization, investigation, formal analysis, data curation, writing-original draft, writing review and editing, and visualization. YL: investigation, validation, formal analysis, data curation, writing-original draft, writing review and editing, and visualization. XD: data curation, formal analysis, validation, and writing – original draft preparation. YK: data curation, validation, and writing review and editing. JL: data curation, writing – original draft preparation, and writing review, editing. WJ: data curation and writing review and editing. PY: data curation and writing review and editing. YW: data curation, formal analysis, and writing review and editing. YD: writing review and editing, visualization, formal analysis, and data curation. JX: conceptualization, investigation, writing review and editing, visualization, supervision, funding acquisition. ASKC: writing review and editing, investigation, and formal analysis.
