# Supplementary material for: Influence of metabolic syndrome and lifestyle factors on thyroid nodules in Chinese adult men: a cross-sectional study
Source: Eur Thyroid J. 2023 Nov 3;12(6):e230168. doi: 10.1530/ETJ-23-0168 (PMC10692680; doi:10.1530/ETJ-23-0168)
Supplement: Supplementary Material 1. Overview of the final observed variables [file supplementary_material.pdf]

Supplementary Material 1. Overview of the final observed variables

Latent variables	Observed variables	Coding	Description
	Age	1: age 19-29	
		2: age 30-29	
		3: age 40-49	
		4: age 50-59	
		5: age 60+	
	Work intensity	1: Non-worker	
		2: Office worker	
		3: Light manual laborer	
		4: Medium and heavy manual laborer	
	Ethnic	0: Han	
		1: Minorites	
	Drinking Coffee	0: No	
		1: Yes	
	Eating habits	0: Eating on time	
		1: Not eating on time	
Substance use	Drinking alcohol	0: No	"Drinking alcohol" and "Smoke" are observed variables of latent variable "Substance use"
		1: Yes	
	Smoke	0: No	
		1: Yes	
	TNs	0: No	
		1: Yes	
	Hypertension	0: No	"Prediabetes" merged with 'Normal'
		1: Yes	
	Diabetes	0: No	
		1: Yes	
	Dyslipidemia	0: No	
		1: Yes	
	Centripetal obesity	0: No	Using centripetal obesity as a grouping criterion
		1: Yes	
	MetS	Hypertension + Diabetes + Dyslipidemia + Centripetal obesity	Number of symptom of MetS

MetS: Metabolic Syndrome
